# Re: Assessment of sexual functions in partners of women with complaints of urinary incontinence

**DOI:** 10.1590/S1677-5538.IBJU.2016.0647

**Published:** 2017

**Authors:** Mustafa Kadihasanoglu, Emin Özbek

**Affiliations:** 1 Department of Urology, Istanbul Training and Research Hospital, Istanbul, Turkey

*To the editor,*

We have read the article by Keles et. al. (1) which was an interesting study evaluating the sexual function of partners of women with complaints of urinary incontinence (UI). In this study, they tried to assess the sexual function in men with female partners suffering stress or urge UI by International Index of Erectile Function (IIEF). They showed that erectile function in the partners of women with UI may be adversely affected by the UI of their partners.

Multifactorial nature of sexuality should be considered during the evaluation of the sexual function of men and women. The factors affecting male sexual dysfunction are depression, anxiety, stress, other mental health problems and physical causes including diabetes, obesity, metabolic syndrome, cardiovascular diseases, hypertension, treatments for prostate cancer, benign prostate hyperplasia, neurological diseases, hypogonadism, smoking, and pelvic surgeries (2). Although this study did not evaluate the sexual function of women, the UI has a negative impact of sexual function of the women (3). A significant correlation between female sexual function and male erectile function has been reported, based on scores from the Female Sexual Function Index (FSFI) and IIEF (4). In addition to these factors, several andrologic studies suggested that total testosterone plays an imperative role in erectile physiology in humans and its deficiency causes ED. The recent EMAS study found that total testosterone is significantly associated with ED (5).

Thus, we consider that these factors for male dysfunction, as mentioned above, are limitations of this study, because the authors did not evaluate these factors and female sexual function and they aimed to reach a conclusion that UI in women have adverse effect on male sexual function. In addition, the authors did not report the hormonal status of male partners and if they use were using any medical treatment including hormones, and phosphodiesterase inhibitors, because these medications can affect sexual function. As a result, we claim that these factors should be indicated as a limitation to strengthen the outcomes of the study.
